# Impact and burden of acid sphingomyelinase deficiency from a patient and caregiver perspective

**DOI:** 10.1038/s41598-021-99921-6

**Published:** 2021-10-25

**Authors:** Robin Pokrzywinski, Asha Hareendran, Luba Nalysnyk, Sandy Cowie, Joslyn Crowe, Justin Hopkin, Dhaivat Joshi, Ruth Pulikottil-Jacob

**Affiliations:** 1 Evidera, 7101 Wisconsin Ave, Suite 1400, Bethesda, MD 20814 USA; 2 Evidera, 201 Talgarth Rd, Hammersmith, London, W6 8BJ UK; 3 Sanofi Genzyme, 50 Binney St., Cambridge, MA 02142 USA; 4 International Niemann-Pick Disease Alliance, Suite 2, Vermont House, Concord, Washington, Tyne and Wear, NE37 2SQ1 UK; 5 National Niemann-Pick Disease Foundation, Inc., 401 Madison Avenue Suite B, Fort Atkinson, WI 53538 USA; 6 Rare Diseases, Sanofi Genzyme, Thames Valley Park, Reading, UK

**Keywords:** Metabolic disorders, Outcomes research

## Abstract

Acid sphingomyelinase deficiency (ASMD), historically known as Niemann–Pick disease (NPD) types A, A/B, and B, is a rare, progressive, potentially fatal lysosomal storage disease with a spectrum of phenotypes. Little is known about how ASMD symptoms affect the lives of patients and their caregivers. In a cross-sectional qualitative study conducted in the US and UK, and in collaboration with the National Niemann–Pick Disease Foundation (US) and Niemann–Pick UK, we investigated the symptom experience of patients with ASMD types B and A/B and explored how the disease impacts their and their caregivers’ lives. The study included 17 adult patients (mean age 38.7 years, 12 female), three caregivers of adults with ASMD, 12 pediatric/adolescent patients with ASMD (mean age 10.5 years, six female), and 12 caregivers of pediatric/adolescent patients with ASMD. The most commonly reported disease manifestations were respiratory (n = 26, 89.7%), abdominal (n = 25, 86.2%), and musculoskeletal symptoms (n = 23, 79.3%); excessive bleeding or bruising (n = 20, 69%); fatigue (n = 20, 69%); gastrointestinal symptoms (n = 18, 62.1%); and headache (n = 15, 51.7%). ASMD was reported to negatively impact patients’ physical function (n = 23, 79.3%), self-esteem (n = 18, 62.1%), emotions (n = 16, 55.2%), social function and relationships (n = 16, 55.2%), and personal care (n = 9, 31%). Providing care for individuals with ASMD negatively affected caregivers’ emotional well-being (n = 12, 80%), social function (n = 4, 26.7%), relationships (n = 6, 40%), and financial security (n = 7, 46.7%). The physical toll of providing care, the need for lifestyle changes, and the responsibility for making medical decisions added to the burden for caregivers. Alternatively, some caregivers noted that caring for a loved one enhanced their spirituality, providing them with a different outlook on life and a deeper personal resolve. This study showed that ASMD is a substantial burden for patients and caregivers, with long-term physical, emotional, social, and financial impacts. The study confirmed commonly known manifestations of ASMD, especially respiratory problems, but also identified less recognized ones, such as dermatological complications. The data collected and insight gained from this study should enhance clinical care, help evaluate new treatments, and inform health care decision making for patients with ASMD.

## Introduction

Acid sphingomyelinase deficiency (ASMD), historically known as Niemann–Pick disease (NPD) types A, A/B, and B, is a rare, progressive, potentially fatal lysosomal storage disease caused by pathogenic variants in *SMPD1*, the gene encoding acid sphingomyelinase^1^. ASMD has an estimated incidence of 0.4 to 0.6 per 100,000 births worldwide, although it might be underestimated due to the rarity of the disease and poor disease awareness^2,3^.

ASMD is a disease spectrum divided into three types: type A, also known as infantile neurovisceral ASMD; type A/B, also known as chronic neurovisceral intermediate ASMD; and type B, also known as chronic visceral ASMD^3,4^. ASMD type A is associated with rapidly progressing visceral manifestations and neurodegeneration, which typically lead to death by the age of 3 years, most often due to respiratory failure. Variable visceral manifestations also occur in ASMD types B and A/B, and neurological manifestations are rare in ASMD type B, but mild to severe neurological manifestations occur in ASMD type A/B, although they progress more slowly than in ASMD type A^5^. The most common manifestations in ASMD types B and A/B include interstitial lung disease, hepatosplenomegaly, thrombocytopenia, dyslipidemia, delayed growth, shortness of breath, fatigue, and pain^6–9^, and many patients develop life-threatening complications, due mostly to hepatic and pulmonary disease^1,8,10^.

Although several studies have explored the clinical manifestations of ASMD, how the disease impacts patients and their caregivers is not well known. A qualitative study published in 2009 of eight adolescent and adult patients and nine parents of adolescent patients provided some insight into the psychosocial effects and the financial impact of type B ASMD^11^. In the current study, we further investigated the symptom experience of patients living with ASMD types B and A/B and explored how the disease impacts the lives of patients and their caregivers.

## Methods

### Study design

This was a cross-sectional qualitative study using grounded theory conducted in the US and UK between August 2015 and December 2019. In-depth interviews were conducted in English in person or by phone. For pediatric/adolescent patients (aged < 18 years), caregivers had to be present. Pediatric/adolescent patients > 8 years of age were interviewed with the help of their caregivers, when needed. For patients < 8 years of age, the caregiver was interviewed in place of the patient. Additionally, interviews of caregivers without the patient present were conducted to explore how ASMD affects them. This study was approved by Advarra Institutional Review Board (Columbia, MD, USA). All participants provided informed consent or assent to participate in the study. The results of this study are reported in compliance with the Consolidated Criteria for Reporting Qualitative Research guidelines^12^.

### Participant recruitment

The study enrolled pediatric/adolescent (< 18 years) and adult (≥ 18 years) patients with ASMD type B or A/B and primary caregivers (i.e., live with the patient ≥ 6 months) of patients with ASMD type B or A/B. Participants were recruited through announcements, website postings, newsletters, social media sites, and patient conferences by the National Niemann–Pick Disease Foundation (US) and Niemann–Pick UK. All participants had to be capable of completing the interview with or without the additional help of their caregivers in the investigator’s opinion.

### Data collection

All interviews were conducted by researchers experienced in qualitative research methods (Supplemental Table 1). Participants did not have prior relationships with and were not informed about the interviewers prior to study commencement. Only the interviewer, participant, and caregiver for pediatric/adolescent patients were present during the interview. For pediatric/adolescent patients (aged < 18 years), caregivers had to be present.

To facilitate the interviews, semi-structured interview guides were developed based on a targeted literature review and interviews with clinical experts. Interviews were conducted at ASMD patient and family conference facilities within the US and UK or by telephone. All pediatric/adolescent participants were interviewed in person. All interviews were conducted in two sessions totaling approximately 60–120 min with a break in-between, if needed.

Patient interviews started with questions tailored to obtain an overview of the patient’s disease history, including the nature of disease onset and the pathway to diagnosis, followed by a detailed discussion of the patient’s experiences with ASMD symptoms. After this initial discussion, the participant was asked to describe how ASMD symptoms affected their daily functioning and quality of life.

Caregivers were initially interviewed alone about the person for whom they cared. Next, the caregivers were prompted to obtain spontaneous responses about how ASMD impacted them, and they were probed about suspected areas of impact to the person for whom they cared.

At the end of each interview, participants completed a demographic and clinical questionnaire. Interviews were audio-recorded and transcribed. Data saturation, the point at which no new information is uncovered^13^, was documented but not discussed with the participants. Transcripts were not returned to participants for comment or correction.

### Data analysis

Participant data were de-identified for analysis. Data collected for adult and pediatric/adolescent patients with ASMD were analyzed separately. Data from the qualitative interviews (transcripts) were qualitatively analyzed using a content analysis approach. Analyses were guided using coding dictionaries that were developed to organize and systematically categorize the text in the interview transcripts using the qualitative analysis software program ATLAS.ti version 7.5.1 (Scientific Software Development GmbH, Berlin, Germany). Data were coded by at least two researchers. Data were examined for general themes, specific concepts, issues, and concerns associated with ASMD symptoms and impacts by participant type (patient or caregiver), with a focus on words or phrases that participants used to describe symptoms and impacts associated with ASMD or, for caregivers, used to describe caring for someone with ASMD. Concept-tracking grids were developed to assess concept saturation, the point at which no substantially new themes, descriptions of a concept, or terms were introduced as additional interviews were conducted^14^. Participants did not provide feedback on the findings.

### Ethics approval and consent to participate

IRB/Ethics approval was obtained through the Advarra (Columbia, Maryland, US), Pro00012510, May 2015 (under former IRB name, Chesapeake Institutional Review Board).

### Informed consent

All participants provided informed consent to participate in the study. Written informed consent was obtained from caregivers or legal guardians for participants under the age of 16 years old.

### Consent for publication

Individual patients are not identified in the publication.

## Results

### Participants

Between August 2015 and December 2019, 17 adult patients (≥ 18 years), and three caregivers of adult patients living with ASMD, 12 pediatric/adolescent patients (< 18 years) and their caregivers were enrolled. All participants completed the study.

#### Patients

Adult patients had a mean age of 38.7 years (range 18–62), and most were female (n = 12/17) and White (n = 16/17). Pediatric/adolescent patients included equal numbers of males and females (n = 6/12 each), and the mean age was 10.5 years (range 2.4–17.0 years). Data on race were available for only four pediatric/adolescent patients, of whom two were White and two Asian.

Most adult (n = 14/17) and pediatric/adolescent patients (n = 9/12) identified as having ASMD type B (Table 1). Two adult patients and one pediatric/adolescent patient identified as having ASMD type A/B. Disease type was unknown for one adult (who self-reported severity as mild) and two pediatric/adolescent patients. No data on neurological disease and rate of disease progression were available for these patients. However, the two pediatric/adolescent patients with unknown disease type were ages 12 and 13, so were presumed to be either ASMD type A/B or B.

**Table 1:** Demographic and clinical characteristics of patients included in the study.

Characteristic	Adult patients	Pediatric/adolescent patients
	(N = 17)	(N = 12)
**Age (years)**		
Mean (SD)	38.7 (14.5)	10.5 (4.7)
Range	18.0–62.0	2.4–17.0
**Gender, n**		
Male	5	6
Female	12	6
**Race/ethnicity, n**		
White	16	2
Asian	0	2
Other	1^a^	0
Not collected	0	8
**Type of ASMD, n**		
Type B	14	9
Type A/B	2	1
Missing	1	2
**Age at ASMD diagnosis (years)**		
Mean (SD)	14.3 (16.7)	2.0 (1.1)
Median [range]	4.0 [0.5–42.0]	1.8 [0.8–3.5]
**Age at onset of ASMD symptoms (years)**		
Mean (SD)	6.6 (8.4)	1.7 (1.0)
Median [range]	2.0 [0.0–24.0]	1.5 [0.5–3.0]
**ASMD severity**^**b**^** (n, %)**		
Mild	4	–
Moderate	10	–
Severe	2	–
Very severe	1	–
Missing	0	–

*ASMD* acid sphingomyelinase deficiency, *SD* standard deviation.

^a^Other reported race as White and Asian.

^b^Adult patient self-report; pediatric/adolescent patients did not self-report severity.

Severity of ASMD was mostly mild or moderate as self-reported by adult patients (n = 14) and as self-reported by or reported by caregivers for pediatric/adolescent patients (n = 7). A few patients had severe (2 adult patients, 2 pediatric/adolescent patients) or very severe (1 adult patient) ASMD. Caregiver-reported ASMD severity for three pediatric/adolescent patients are missing.

Adult patients started experiencing symptoms at a mean age of 6.6 (range 0.0–24.0) years and having been diagnosed at a mean age of 14.3 (range 0.5–42.0) years. Pediatric/adolescent patients started experiencing symptoms at a mean age of 1.7 (range 0.5–3.0) years and were diagnosed at a mean age of 2.0 (range 0.8–3.5) years.

#### Caregivers

Caregivers of adult patients had a mean age of 57.7 years (range 47.0–65.0), whereas caregivers of pediatric/adolescent patients had a mean age of 38.4 years (range 33–45 years), (Table 2). Of the 13 caregivers for whom data were available, most were female (n = 11), White (n = 11), and were caring for patients with ASMD type B (n = 11).

**Table 2:** Demographic characteristics of caregivers of patients with ASMD included in the study.

Characteristic	Caregivers of pediatric/adolescent patients	Caregivers of adult patients
	(N = 12)	(N = 3)
**Age (years)**		
Mean (SD)	38.4 (4.0)	57.7 (9.5)
Range	33.0–45.0	47.0–65.0
**Gender, n**		
Male	1	1
Female	9	2
Missing	2	0
**Race/ethnicity of patient or caregiver, n (%)**		
White	8	3
Asian	1	0
Mixed—White and Asian	1	0
Not collected	0	0
Missing	2	0
**Type of ASMD of patient, n**		
Type B	9	2
Type A/B	1	1
Missing	2	0
**Caregiver assessment of patient’s ASMD severity (n, %)**		
Mild	3	1
Moderate	4	2
Severe	2	0
Very severe	0	0
Missing	3	0

*ASMD* acid sphingomyelinase deficiency, *SD* standard deviation.

### ASMD symptom experience

**Figure 1:**
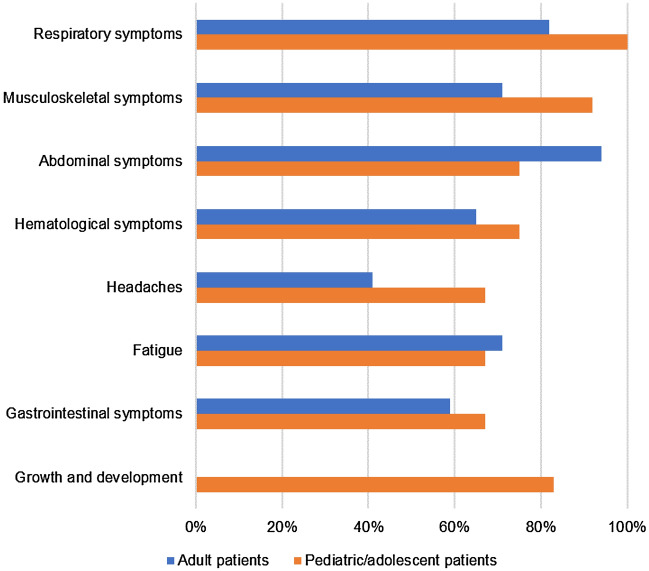
Symptoms reported by patients with acid sphingomyelinase deficiency. “Growth and development” is not shown for adult patients because it was not relevant.

Patients reported a wide range of manifestations, most frequently respiratory (n = 26, 90%), abdominal (n = 25, 86%), and musculoskeletal (n = 23, 79%) symptoms (Fig. 1). Other reported manifestations were excessive bleeding or bruising (n = 20, 69%), fatigue (n = 20, 69%), gastrointestinal symptoms (n = 18, 62%), and headache (n = 15, 52%).

#### Respiratory symptoms

All pediatric/adolescent patients (n = 12) and 14 of 17 adult patients reported respiratory symptoms, including shortness of breath, cough, repeated respiratory infections, chest pain, and difficulty breathing. Patients described a high level of severity, which, in addition to physical discomfort, resulted in other physical limitations. One adult patient explained:

*“…every time I have to go cycling to school, and when I come to school, I’m like [panting] like I just have to…stand there for a minute and do nothing just to find my normal breathing again…There was one day I had to go babysitting and I have to…get up a hill and not down and it was like it was a really big—I couldn’t breathe anymore and I was like going down the hill and I was like not having like short breaths because it was really painful, my chest here.” [Patient A]*

Patients also described the progressive nature of ASMD through their experiences with respiratory manifestations. For example, one adult patient explained:

*“…I’ve noticed within the last two years my breathing has gotten a lot worse to where I can just walk minimal, like from here to the lobby, and I’m already out of breath…I am being told now by my pulmonologist that I have to be on oxygen if I’m doing any amount of walking…I have a little portable concentrator that I carry with me that—just like getting through the airport, from off the plane to where the shuttle picked us up to come here, I was completely out of breath, I mean I literally had my oxygen on and still had to take my time and get through it.” [Patient B]*

#### Abdominal symptoms

Various abdominal symptoms were reported by 16 of 17 adult patients and 9 of 12 pediatric/adolescent patients. This included abdominal pain/discomfort and an enlarged or distended abdomen that sometimes caused pain while bending due to abdominal distention. Patients described the experience with a range of severity and as focal or diffuse. For example, one adult patient stated:

*“I’ve still got a distended abdomen. I know that there are enlarged organs, I don’t know what they are, but I can feel if I’m lying on my back, I can feel…some things aren’t quite as they should be.” [Patient C]*

Another adult patient explained:

*“It’s usually right under the rib cage, it’s usually on the spleen side. I do get it on the liver side sometimes, but mostly the spleen, um, it will just be like a real tight cramping and sometimes I get throbbing pains—and sometimes I end up having to take medicine to help me sleep because I can’t deal with it.” [Patient D]*

Several other patients described secondary sleep disturbance due to abdominal distention and related pain. An adult patient described that pain from the disease disrupted their sleep:

*“I have lost sleep because I was in pain and especially because, uh, not just the liver, but just in general abdominal pain...it’s just—it’s uncomfortable. As much as I don’t like the pain, it’s just uncomfortable. That’s why I can’t sleep.” [Patient F]*

#### Musculoskeletal symptoms

Musculoskeletal symptoms, including muscle weakness, bone pain, and joint pain, were reported by 12 of 17 adult and 11 of 12 pediatric/adolescent patients. Although musculoskeletal symptoms were reported by both patient cohorts, only adult patients reported back pain, focal pain in the extremities, and muscle cramps, whereas only pediatric/adolescent patients described experiencing poor muscle tone, joint contractions, joint pain, and weak bones. Descriptions by adult patients indicated that these symptoms develop and progress throughout the patient’s lifetime. One adult patient described bone pain as an important symptom:

*“Bones…especially when I was a kid, I had really, really bad bone pain, and I still get it….I know it’s bone, I know it’s not muscular.” [Patient G]*

Another adult patient reported muscle and joint problems:

*“I thought my top one would be, uh, my muscle and my joint problems because that can—that can reflect on what I’m doing. …I experience this a lot during secondary school. If—if I have an issue with my—my leg, or my knee, or something I cannot walk. Um, so I—I’d be down for a good 20 minutes just waiting for it to pass which is kind of the worst bit which the thing is you just have to wait for it to pass and there’s nothing much you can do.” [Patient F]*

Pediatric/adolescent patients also described knee pain. The caregiver of one adolescent patient explained:

*“She complains when…she walks, her knees are hurting constantly.” [Mother of Patient E]*

An adolescent patient described:

*“My knees are like really bad. Yeah, because when I had to run through the hall, it just gets—it really hurts. That was this week. “Your legs and your knees you said?” Yeah. This—this is the first time I had like that problem with my knees. It was weird, but—but most my—my legs.” [Patient A]*

#### Other common symptom themes

Both adult and pediatric/adolescent patients reported fatigue, excessive bleeding and bruising, abdominal pain, gastrointestinal symptoms, and headaches. The intensity of fatigue and its effect on normal daily activities was highlighted. For example, the caregiver of a pediatric patient explained their child’s fatigue:

*“The tiredness is just when he has a bad day really, he could wake up one morning and be a bit tired for the day or he could just be doing too much and get too tired and—and ache and just want to sit down generally and just not do what the others are doing. He’ll want to, you know, but you can tell that he—he doesn’t have the energy to do it anymore throughout the day or he has to be told by us to sit down and—and just to calm down a bit.” [Father of Patient H]*

One adolescent patient explained it in their own words:

*“Sometimes I get like exhausted. I get really tired by the end of the day. It’s hard for me to—to do some things like—like taking my socks off or just basic things, like that. I get over tired and then my mom has to help me do things like take off my socks and um put me into bed and stuff like that, just little—very basic things that I need help with.” [Patient I]*

Many patients reported gastrointestinal involvement within their overall symptom experience. In addition to single gastrointestinal symptoms, such as stomach upset, nausea, vomiting, and diarrhea, many described a confluence of gastrointestinal symptoms. For example, one adult patient described difficult bowel movements:

*“I do have very bad bowel movements, it’s basically—it’s very watery whenever I have to go to the bathroom. I can’t hold it very well, um, I would say, like, I have irritable bowel syndrome just because of the fact that I have to run to the bathroom, it’s not something I can really control, and I know even throughout school my teachers are why do you always have to go to the bathroom… It’s kind of embarrassing. When I was little I used to think who’s going to want to date me, how’s that going to be on a date whenever I have to rush to use the restroom or something, but I’m very fortunate that my husband is very understanding and everything.” [Patient D]*

For one adolescent patient, bouts of incontinence due to problems with gastrointestinal motility and satiety sometimes resulted in an inability to enjoy food:

*“I always love to eat, but when I eat too much it makes me—it makes me sick and I usually have to go to the bathroom at least three times afterwards. That’s one of the biggest symptoms too. I love to eat and then overeat and everything gets overcrowded in my stomach and I usually have to use the bathroom at least two to three times until my stomach gets better.” [Patient I]*

Other symptoms such as headaches and bruising/bleeding were universally described by adults and pediatric/adolescent patients. An adult patient described experiences with bleeding:

*“…when I was 6 is when they found out I had the bleeding problem, because they went in to do a simple tonsillectomy and I ended up almost bleeding to death three different times, because they couldn’t get the bleeding to stop. So, that’s when I learned that I did have a serious issue with bleeding and surgeries, so I had to be really careful from then on out.” [Patient B]*

Parents of patients also expressed concern about the hematological aspects of the disease. For example, some parents were concerned about their child bruising easily because it was not always clear how the bruises arose. When asked about what symptoms are most concerning, the caregiver of one pediatric patient said:

*“I’d say the bruises because he does, um, pick up bruises and some of them are quite I mean large.” [Father of Patient J]*

When asked, “Does she ever have any bruises that she doesn’t remember getting?”, a caregiver of a pediatric patient responded:

*“Yes, recently she’s had a few and she’s just shown me. And I’ve—I’ve asked her quite a lot, Oh. How did you get that? Because it seems to be quite big and she doesn’t remember bumping it anywhere at school.” [Mother of Patient K]*

Additionally, pediatric/adolescent patients were specifically aware of and expressed concerns about excessive bleeding. When asked, “Do you get nose bleeds or bleed?”, one adolescent patient responded:

*“Yes, okay, this is a big problem with my life. Um, so when I bleed, I—I am low on platelets, so I bleed a lot. When—when it—if I get a little cut, it will bleed for a really long time. It’s really hard for it to stop. Um, two years ago, when I went to summer camp, I got a nosebleed from the dryness and it just wouldn’t stop.” [Patient I]*

Unique to pediatric/adolescent patients were effects on physical growth and development. Symptoms related to physical development were reported for 10 of the 12 pediatric/adolescent patients. This included short stature, growth issues, abnormal facial features, delay in maturity, being underweight for age, and problems with hair texture. Another unique manifestation in pediatric/adolescent patients was dermatological symptoms, reported by 7 of 12, including skin tightness, rashes, and warts. As described by one adolescent patient:

*“Um, sometimes…when I took my clothes off it’s like um, all red dots here…I don’t know how you would call it…it’s like all over my feet. Like not that bad, but I have a lot of on my feet and we went to the doctor for it and I have like really dry legs also…Sometimes it—because I have one on my heel and it’s like—it’s like blocking the way into my shoes, so it—sometimes it hurts and I’ll say oh, no, it hurts...when I’m in my shoe, it’s like it’s okay, but it’s just getting in my shoes that sometimes is a problem.” [Patient A]*

### Impact of ASMD on daily lives of patients

**Figure 2:**
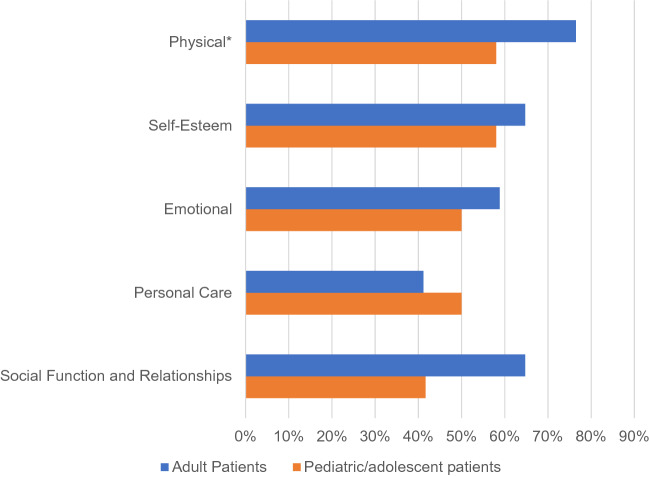
Impact of ASMD on patient’s daily lives. *Adult patients: common daily activities. Pediatric/adolescent patients: hobbies, activities and sports.

Irrespective of age, ASMD was a significant burden for patients. ASMD had a negative physical impact (23 of 29 patients overall), impact on self-esteem (n = 18), emotional impact (n = 16), impact on social function and relationships (n = 16), and impact on personal care (n = 13). Figure 2 displays the areas of impacts on patients.

#### Physical impact

Difficulty in performing common daily activities (household chores, simply walking around the home, and helping with childcare duties) was reported by 12 of 17 adult patients. Most adult patients (13 of 17) reported that, due to the impact of the disease, they were unable to participate in other desired recreational activities and hobbies. Adult patients with ASMD consistently reported feeling that ASMD impacted their ability to exercise and, as a result, were often unable to participate in sports. For example, one adult patient stated:

*“I haven’t been able to play certain sports. I had to quit doing softball because the ball is pitched right towards your abdomen, and I couldn’t do that.” [Patient D]*

Another adult patient explained needing to recuperate after physical exercise:

*“If I do too much…I’m usually in the bed for a few days or you know just trying to recuperate just from um you know if I do something strenuous or you know something that you know um takes a lot out of me. Then it feels like I pay the price for it [laughter] afterwards you know.” [Patient L]*

Similarly, 7 of 12 pediatric/adolescent patients described limitations in playing sports or participating in hobbies or activities that were more physical in nature. In addition to physical exhaustion, they also reported symptoms like abdominal distention and pain that limited their ability to participate in desired activities. One adolescent explained:

*“I have problems with sports or in sports. I’m not allowed to do like contact sports, as I said, and that’s—that’s the problem that I usually have. I can’t do certain things, like when we’re doing the fitness test, I’m encouraged just to try my best and do as many things as I can, but yeah, that’s one of the—that’s a big problem.” [Patient I]*

Pediatric/adolescent patients also reported physical limitations that prevented them from participating fully in school. One caregiver explained that there were days when their child could not attend school:

*“She has been sent home a couple of times [because of the pain]…once she needed the toilet. So, she ended up getting sent home because she didn’t want to go on school toilet. [Laughter] She said she had pain in the stomach.” [Caregiver of Patient M]*

This was also highlighted by adult patients when remembering their younger years as a student. Physical symptoms, such as gastrointestinal manifestations and restricted mobility caused difficulties in maintaining expected school attendance. As a result, securing additional tutoring and access to reasonable accommodations were required to complete assigned schoolwork and maintain other educational commitments.

#### Impact on self esteem

Most adult (11 of 17) and pediatric/adolescent (7 of 12) patients reported that their disease negatively affected their self-worth and self-perception, including body image, feeling being different from peers, and an inability to take part of normal age-appropriate activities. Patients also reported having to choose less desirable but more practical clothing options because of ASMD. An adult patient explained how the disease affected their sense of self-worth and their mood and that they felt like they were letting their children and friends down:

*“…it gives you um a sense of where you don’t feel like yourself—your self-worth is as much as everybody else you know because you’re always letting people down…that affects your mood…because I always feel like I’m letting my kids down or you know my friends down and you know if I have to cancel and you know stuff like that…” [Patient L]*

One adolescent patient said:

*“It makes me feel upset or sad when—when people come up and ask me about it [my disease]. That’s something that I—it doesn’t really affect me, but it affects me when—when people come up and ask me questions about it and why—and I don’t look like everybody else.” [Patient I]*

The caregiver of one pediatric patient explained:

*“He feels that he…looks a bit of a wimp…because he can’t do contact sports, can’t carry his bag, you know, there’s a lot of things he can’t do, so he’s very self-conscious of that.” [Mother of Patient N]*

#### Emotional impact

Overall, 16 of 29 patients reported that ASMD affected their emotions, causing anxiety, depression, sadness, frustration, concern, and fear. More than half (10 of 17) of adult patients reported that ASMD affected their emotions, with some expressing sadness because they felt that they consistently disappointed others. Many patients noted that the disease limited their ability to fulfill obligations or to maintain commitments. Despite these challenges, other adult patients displayed resilience by actively pursuing a positive mentality. For example, one adult patient said:

*“If you allow yourself to get depressed you will shave years off your life and you will increase your propensity for angina or, uh, shortness of breath. It’s just you have to maintain a positive outlook. I’m not positive about the disease, but I’m positive in my ability.” [Patient O]*

In half of the pediatric/adolescent patients, ASMD had an emotional impact, including frustration, irritation with symptoms, sadness, and worry about their condition. Although they used different words to express themselves, they described being different from their peers, feeling self-consciousness about their appearance or having to answer questions about their condition, and being bullied by peers.

The caregiver of one pediatric patient described concern over anxiety and its effect on the child’s well-being:

*“It does affect it negatively, because I don’t think it’s good to be that stressed out all the time or worried.” [Caregiver of Patient P]*

The caregiver of an adolescent patient described bullying by other children:

*“She was actually bullied, um, when she started high school. I don’t know if it’s a girl thing or generally, um, because she’s quite short.” [Mother of Patient K]*

#### Impact on personal care

ASMD impacted personal care in 6 of 12 pediatric/adolescent patients and 7 of 17 adult patients. In these cases, patients needed assistance from caregivers because of pain and limited range of motion. Reported challenges included personal hygiene and grooming, self-dressing, and maintaining a healthy diet.

One parent of an adolescent patient explained:

*“Yeah, he can’t put socks on because it hurts. Um, and I think—he can put his shoes on, but he struggles with socks some reason, I think it’s because with shoes you can kind of put your toe in and push your foot in. But with socks you’ve actually got to get your hand down as far as your toe.” [Mother of Patient N]*

Reports of increased gastric motility, early satiety, and abdominal discomfort were coupled with patient comments about limitations to their ability to eat sufficiently or as much as they desired. One adolescent explained:

*“I just feel like if I eat a lot it’s going to make my stomach hurt, and I wish that I didn’t have that problem because I really like to eat, and I would like to eat as much as I want. Right. When my stomach says stop, I—it just like—it’s telling me to stop.” [Patient I]*

One caregiver described the experience of her daughter:

*“She’s been on the supplement bottles, you know, because at times she stopped. She wasn’t eating and—eating properly. …she was on…food supplement bottles that she would have—she would have two a day.” [Mother of Patient K]*

#### Impact on social function and relationships

Many (11 of 17) adult patients reported that ASMD affected their social lives and relationships with family and friends. Adult patients described limitations involving social activities and frequently cancelling plans and other arrangements. Exhaustion, limited mobility, and frequent diarrhea were cited as causing major disruptions in time with friends. This resulted in patients feeling disappointed about letting others down, embarrassed, and anxious in social settings. An adult patient explained:

*“If you’re trying to do something you know like go have dinner with friends or something and then go to a movie or something afterwards, it’s like I always have to go to the bathroom.” [Patient L]*

Another comment from the adult patient:

*“I’ve had a few friends that would get upset you know because you know have to cancel plans and not being able to keep plans. And so uh and I’ve lost a few friends along the way…I think it just becomes frustrating to other people. [Patient L]*

As with adult patients, physical symptoms had a major impact on the lives of pediatric/adolescent patients. Significant social hardships such as bullying by peers, difficulties interacting with classmates, trouble making friends, and an inability to date or engage in romantic relationships were reported by 5 of 12 pediatric/adolescent patients. One older adolescent patient said:

*“If I’m feeling particularly unwell…I can’t go out...like going out with your flat mates or something like that is quite, uh, an important bonding time with them. It does get a bit frustrating sometimes.” [Patient F]*

The caregiver of a pediatric patient explained her child’s experiences:

*“He’s started to socialize with his own age group, um, very well this last year, but it took a while. Before that he was suspicious of anybody his own age...friends his own age is a little tricky.” [Mother of Patient Q]*

The social impact of ASMD was expressed alongside the emotional toll of the disease. One adult patient explained:

*“You end up feeling like you don’t have much to give because you have only got enough energy to basically keep yourself going and then you feel that you don’t have anything left over for other people.” [Patient R]*

### Impact of ASMD on daily lives of caregivers

**Figure 3:**
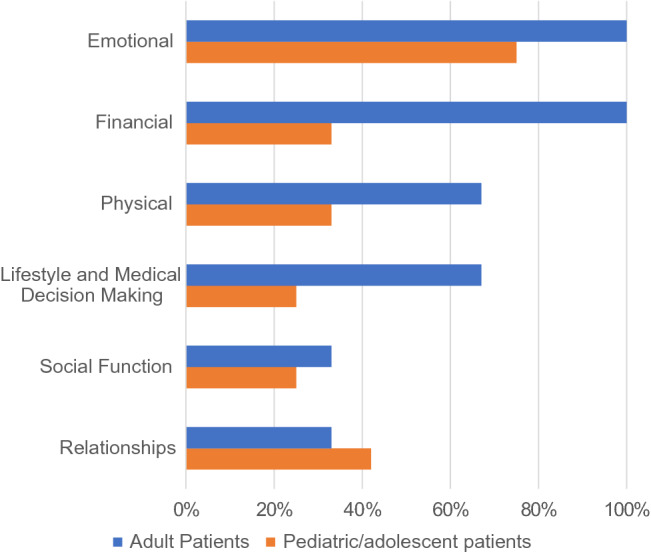
Impact of ASMD on caregivers.

For caregivers, providing care for individuals afflicted with ASMD impacted emotional well-being, social function and relationships, and financial security (Fig. 3). It also was a burden because of the physical toll, the need for lifestyle changes, and the responsibility for making medical decisions. A few caregivers, however, explained that caring for a loved one enhanced their spirituality, providing them with a different outlook on life and a deeper personal resolve. [Caregiver of Patient S].

#### Emotional impact

All three caregivers of adult patients and 9 of 12 caregivers of pediatric/adolescent patients described a variable emotional impact of fulfilling their role as a caretaker. One individual reported a history of suicidal thoughts and attempts. Caregivers reported anxiety, stress, worry, frustration, guilt, exhaustion, and depression. When asked about the daily challenges of caring for a pediatric patient, one caregiver said:

*“Definitely the tiredness…it’s sort of like caring for a toddler…when they’re first born and you’re up every night, and you’re like, okay, there’s a light at the end of the tunnel…But I don’t really get that, because I don’t know if she’s going to progress much further than she is or not, like I don’t know how much worse it can get.” [Caregiver of Patient P]*

A caregiver of a pediatric patient cited “hate, blame, anger, emotion” [Caregiver of Patient C], while another said:

*“Sometimes I’m just tired, I want him to take care of himself, or I feel guilty because I know I should be saying, no, you need to do this yourself, you’re completely capable and this is not an issue with your health, but you never really know when it is and when it isn’t, so there’s like perpetual confusion.” [Caregiver of Patient S].*

Caregivers who were spouses of patients with ASMD reported that the disease affected their personal relationships. This included concerns about losing their partner, a feeling that they had to be “walking on eggshells” when their partner was depressed, and emotions ranging from anger or resentment to sadness for the affected individual. The caregiver of an adult patient explained:

*“…at first I went through a lot of periods of feeling pretty angry and resentful. I felt like I was just carrying the whole load. I think I’ve kind of gotten past that. And it’s frustrating. The whole thing is pretty frustrating, just because we’re not doing as many things as we used to do. Probably pretty common reactions to anybody who has a spouse with a serious chronic disease. Frustration, some resentment, sadness.” [Caregiver of Patient O]*

Caregivers expressed an overall emotional toll from having to deal with the issues alone or without assistance from a stable support structure. The caregiver of a pediatric patient explained:

*“…my friends don’t really understand. Because it's not physically obvious to them what’s going on. They struggle to understand. So, I don’t really get any support, so I don’t really have much social life. I’m just—like doctors’ appointments and other things, it's just a struggle, sort of having to deal with it by myself.” [Caregiver of Patient R]*

#### Financial impact

All three caregivers of adult patients and 4 of 12 caregivers of pediatric/adolescent patients described a substantial impact of ASMD on their financial status and security. Additionally, five caregivers declared that caregiving had directly affected their employment in some way. Contributing factors included increased medical expenses, reduced working hours to support healthcare needs, and not being able to leave an undesirable employment situation for the main purpose of securing health insurance. A caregiver of a pediatric patient said:

***“****I can’t support my children like I’d like to financially. I expect it had a big impact financially. So, I’ve gone from getting quite a decent wage to not getting anything really. It upsets me because I can’t help the children and it's my responsibility to do that.” [Caregiver of Patient R]*

Another caregiver of an adult patient cited “the out-of-pocket medical costs”, stating:

*“…there’s been several things that we’ve paid for out-of-pocket because the insurance either wouldn’t cover or it was just a big—his motorized chair they wouldn’t pay for, so we paid out-of-pocket for that. We paid for his oxygen—one of his oxygen units, his portable oxygen units we paid for…We’ve been able to handle it, but there’s definitely financial implications.” [Caregiver of Patient O]*

Also, a caregiver of a child patient explained having to remortgage because of the disease:

*“I had to remortgage my house three times to support her… So now, I sit with a massive mortgage and I’m unemployed, but you know, that extra would have been really difficult for us.” [Caregiver of Patient C]*

Caregivers reported having to put aside entertainment and other non-essential activities as financial instability became more pressing. In some instances, as a result of unemployment, underemployment, being underinsured, or the retirement of a spouse, other means of financing were sought. One caregiver described the financial burden related to the volume of medical procedures his wife needed lately, her chronic medication, and her recent retirement:

*“We certainly have less disposable income than we otherwise would have had.” [Caregiver of Patient I]*

Caregivers often cited instances of having difficulty in their jobs while attempting to provide care, such as conflicts with supervisors, which made it difficult to hold a position. The caregiver of a pediatric patient stated:

*“I had a manager that I worked for, and…he couldn’t understand that when [Name] contacted me from university and she was unwell, for me to fetch her….He couldn’t understand that she sometimes needed my support.” [Caregiver of Patient C]*

Another caregiver of a pediatric patient said:

*“…I’d love to leave my job, but I don’t dare because I don’t dare risk losing my health insurance…” [Caregiver of Patient S]*

#### Physical impact

Two caregivers of adult patients explained that caring for a patient with ASMD affected their own physical health, including reduced ability to sleep, exhaustion from arranging and attending frequent medical visits, and interruptions to daily routines.

Four of the caregivers of pediatric/adolescent patients noted feeling tired much of the time as a result of sleeping less or receiving inadequate sleep, as well as difficulties performing activities requiring physical exertion. Similarly, four caregivers of pediatric/adolescent patients described feelings of perpetual tiredness and, in some cases, back pain resulting from carrying the child. The caregiver of one pediatric patient explained the impact of caregiving on their sleep:

*“A lot of it's to the fact that I have daughter with Niemann–Pick just being up, because she’s up and—well, many nights.” [Caregiver of Patient R]*

A caregiver of an adult patient said:

*“…sometimes I’ll have to be up later than I’d like with her and that affects my sleep, which is not good, but it’s pretty trivial.” [Caregiver of Patient I]*

#### Impact on lifestyle and medical decision making

Caregivers described a daily impact of caregiving on their lifestyle. Household responsibilities and the need to make important medical decisions limited caregivers’ ability to participate in personal hobbies or to make time for recreation. Caregivers of two adult patients cited an increased number of chores and other extra responsibilities, such as preparing food or following specialized diets.

Three caregivers of pediatric/adolescent patients described negative effects resulting from the need to home school their child because of bullying or frequently missing school to attend medical appointments and limiting opportunities.

Some caregivers selflessly sought help for the person they cared for but were less apt to seek help for themselves. Stressors from the responsibility of decision making and administering treatments weighed heavily upon the caregiver experience, although a sense of relief was expressed by the caregiver of a pediatric patient when support became available from the advocacy group:

*“Okay, so when it comes to me, I don’t ask for help. However, they have offered it to me many times.…But when it comes to my daughter, then I will call them, for example, if they needed—when she was at school, go to school, you know, talk to the school so they understand…” [Caregiver of Patient C]*

#### Impact on social function and relationships

Caregivers reported that caretaking activities affected their social encounters and relationships. One caregiver of an adult patient (spouse) explained that they were unable to go out as they would like and felt less social, which negatively affected their relationship and intimacy:

* “Well, it’s—I feel sad. We used to go out to dinner—go out to eat 2-3 times a week, go out and catch a movie. Going out to eat has decreased, and I can’t remember the last time we went to a movie.” [Caregiver of Patient O]*

Some caregivers of pediatric/adolescent patients reported similar experiences. In some cases, caregivers reported that the burdens placed on them by the need to care for their child also strained existing relationships. For example, the caregiver of one pediatric patient [Caregiver of patient P] explained that taking their child out of school and caring for her the whole day affected her social life “negatively” and *“*doesn’t leave much room for a social life.” Another said:

*“…it’s occasionally made relationships more difficult because you have a burden, you have a grief and a financial and a time burden kind of on you, and sometimes people think I’m testy, [laughter] and I can see why, but it impairs relationships to an extent.” [Caregiver of Patient S]*

A common theme was that caregiving limited the ability of caregivers to engage in social activities or with friends and family, reducing their social network and support structure. Conversely, caregivers were able to obtain relief from patient advocacy organizations and close family members who were actively involved or provided support with patient care duties. A caregiver of a pediatric patient stated:

*“Niemann–Pick UK have been very good…And the fact that something was there 24 hours as well and people that were in the same position it’s definitely—it’s great that that’s there. And [sigh] immediate family was only my father. He’s at the end of the phone if I need him kind of thing…And there’s the childcare workers in the playschool as well…” [Caregiver of Patient E]*

## Discussion

The present qualitative study provided holistic insight into the experience of patients with ASMD and their care- givers; irrespective of age, ASMD has a substantial impact on patients and their caregivers. Patients with ASMD suffer from a wide range of symptoms across multiple organ systems, most commonly, respiratory, abdominal, and musculoskeletal manifestations, although excessive bleeding or bruising, fatigue, and gastrointestinal symptoms were also frequently reported. As the symptoms worsened, the reported impact of the disease on patients’ daily lives increased. These experiences of symptoms reported by patients are in keeping with previous findings about the natural history and progression of ASMD^3^.

Most importantly, this study provided insight into the numerous areas of daily life affected by ASMD. At all ages, ASMD substantially impacted patients’ self-esteem, emotional health, social function, and relationships. Although some distinct themes were identified, patients often described overlapping and interacting experiences. For example, for pediatric/adolescent patients, ASMD limited the ability to play sports, participate in recreational activities, and socialize. Similarly, for adult patients, severe fatigue prevented them from participating in social functions with friends and colleagues and interfered with the ability to enjoy entertainment or socialize over dinner. Gastrointestinal symptoms and frequent need to use the toilet also had a substantial impact on patients’ lives, disrupting school, complicating social activities like dating, interfering with the ability to enjoy food, and causing embarrassment. Patients also described “letting people down” or “causing disappointment” to others, which resulted in sadness, depression, and decreased feelings of self-worth. ASMD is a complex disease that affects every organ system. These results confirm that ASMD patients can experience a wide variety of symptoms related to their disease, and it is this interplay of multiple symptoms that result in the physical and emotional burdens outlined in this study.

These results expand on previous findings by Henderson and colleagues^11^, who examined the psychosocial experiences of five adolescent and four adult patients with ASMD type B and their families. Like the current study, they identified psychosocial stressors caused by limitations in physical activity and social isolation, as well as a range of other psychosocial issues, including difficulties in psychosocial adjustment, conflicts related to relationships and intimacy, ego integrity, and despair. Although the previous findings were published more than a decade ago, there has been no significant change in the standard of care for ASMD patients. The cumulative research continues to show unmet medical need for ASMD patients based on symptom experience and the negative impact of the disease on patients’ lives.

The current study also provided insight and important information about how ASMD impacts caregivers. Caring for someone with ASMD negatively affected caregivers’ emotional well-being, participation in social functions, relationships, and financial security. As with patients, exhaustion and pain from physical exertion limited the willingness and capacity of caregivers to socialize, and the combination of impacts had an emotional toll on the caregiver. Some caregivers reported being less likely to seek help for themselves than for their loved one. Some further reported staying in jobs they wanted to leave for the sole purpose of securing health insurance, and more than one caregiver reported remortgaging their home to improve their financial security. Despite these burdens, some caregivers explained that caring for someone with ASMD positively affected their spirituality and their overall outlook on life.

This investigation provided an opportunity to identify common themes related to symptom experience and impacts. However, a potential limitation is that it did not assess the relative importance or frequency of individual symptoms and impacts. Another potential limitation is that due to the rarity of ASMD, the sample size was small, so that the population may not be fully representative of the broad spectrum of ASMD experiences. Some patients were recruited through patient and family conferences, which may have biased the sample towards milder disease because they were healthy enough to travel; on the other hand, some patients may have been involved with the advocacy group because of having more severe disease. Therefore, the generalizability of the sample is challenging to characterize. Furthermore, because the study mainly included data from patients and caregivers of patients with ASMD type B, which presents with no or mild neurological symptoms, neurological symptoms and impact may be underreported.

## Conclusions

ASMD is a substantial burden for patients and caregivers, with long-term physical, emotional, social, and financial impacts and with broader manifestations than previously recognized. The information collected in this study should help clinicians, health care decision makers, patient advocates, and patients with ASMD select treatments and develop disease management plans that address patients’ holistic needs.

## Supplementary Information

Supplementary Table 1.

## Data Availability

Qualitative data from transcripts and case report forms will not be shared.

## Abbreviations

ASMD: Acid sphingomyelinase deficiency
NPD: Niemann–Pick disease
