# Supplementary material for: Impact and burden of acid sphingomyelinase deficiency from a patient and caregiver perspective
Source: Sci Rep. 2021 Oct 25;11:20972. doi: 10.1038/s41598-021-99921-6 (PMC8546120; doi:10.1038/s41598-021-99921-6)
Supplement: Supplementary file 1 — Supplementary Table 1. [file 41598_2021_99921_MOESM1_ESM.docx]

**Supplementary material**

**Impact and Burden of Acid Sphingomyelinase Deficiency from a Patient and Caregiver Perspective**

Robin Pokrzywinski^1^, Asha Hareendran^2^, Luba Nalysnyk^3^, Sandy Cowie^4^, Joslyn Crowe^5^, Justin Hopkin^5^, Dhaivat Joshi^3^, Ruth Pulikottil-Jacob^6*^

**Supplemental Table 1. Interviewers**

| **Name** | **Degrees** | **Occupation** | **Gender** | **Training** |
| --- | --- | --- | --- | --- |
| Asha Hareendran | MA, PhD | Senior Research Leader, Outcomes Research Group, Evidera | F | Psychologist with over 20 years of experience in the field of health outcomes research. MA in psychology, human physiology and statistics  4 years of postgraduate training in social work in medical and psychiatric settings  PhD in Quality of Life Research in Cardiovascular Diseases at the National Medical and Mental Health Institutes in India. |
| Milenka Jean-Baptiste* | MPH | Research Associate III, Outcomes Research Group, Evidera | F | Bachelor of Arts degree from the University of Pennsylvania in Philadelphia  Master of Public Health degree from Emory University |
| Andrea Schulz | MA | Research Associate III, Outcomes Research Group, Evidera | F | BA in economics and BA in government and politics, University of Maryland  Master’s degree in integral economic development policy from The Catholic University in Washington, D.C |
| Ella Brookes | BSc | Research Associate I, Outcomes Research Group, Evidera | F | BSc in psychology from Kingston University  Currently studying for an MSc in public health at the London School of Hygiene and Tropical Medicine |
| Owen Cooper | DPsych | Research Associate III, Outcomes Research Group, Evidera | M | DPsych in health psychology from City University of London  MSc in health psychology from Aston University  BSc in psychology with sociology from Nottingham Trent University. |
| Anne Skalicky | MPH | Research Scientist, Outcomes Research Group, Evidera | F | Over 15 years of prior experience designing and implementing a wide range of qualitative and quantitative research projects  Master’s degree in public health (with concentrations in epidemiology and health services) from Boston University  Bachelor’s degree in anthropology from the University of Massachusetts, Boston |
| Savita Bakhshi Anand | PhD | Senior Research Associate, Outcomes Research Group, Evidera | F | PhD in health and social psychology from London Metropolitan University  MSc in health psychology from the University of London  BSc (Hons) in psychology from the University of Reading  Chartered psychologist, chartered scientist, and associate fellow of the British Psychological Society. |
| Peter Chongpinitchai | MBS | Research Associate III, Outcomes Research Group, Evidera | M | Master’s degree in biomedical sciences from Geisinger Commonwealth School of Medicine  Bachelor of science degree in health sciences with biology and chemistry minors from Gettysburg College |
| Anna Steenrod* | MPH | Research Associate III, Outcomes Research Group, Evidera* | F | MPH, Epidemiology and International Health, Boston University  BA, Biology, University of Virginia |
| Huda Shalhoub* | PhD | Senior Research Associate Outcomes Research Group, Evidera* | F | PhD, Psychology Research, Brunel University  MA, Applied Sociology, University of Massachusetts Boston  BA, Sociology, Concordia University |

*Former employee
